# Anxa2 binds to STAT3 and promotes epithelial to mesenchymal transition in breast cancer cells

**DOI:** 10.18632/oncotarget.5199

**Published:** 2015-08-15

**Authors:** Tong Wang, Jie Yuan, Jie Zhang, Ran Tian, Wei Ji, Yan Zhou, Yi Yang, Weijie Song, Fei Zhang, Ruifang Niu

**Affiliations:** ^1^ Tianjin Medical University Cancer Institute and Hospital, National Clinical Research Center for Cancer, The Key Laboratory of Breast Cancer Prevention and Therapy, Ministry of Education, Key Laboratory of Cancer Prevention and Therapy, Tianjin, PR China

**Keywords:** Anxa2, breast cancer, epithelial–mesenchymal transition, epidermal growth factor receptor

## Abstract

Overexpression of annexin A2 (Anxa2) is correlated with invasion and metastasis in breast cancer cells. In this study, breast cancer patients with upregulated Anxa2 exhibited poor overall and disease-free survival rates. Anxa2 expression was also positively correlated with the expression of epidermal growth factor receptor (EGFR) and epithelial–mesenchymal transition (EMT) markers in breast cancer tissues and cell lines. Moreover, knockdown of Anxa2 impaired EGF-induced EMT, as well as the migration and invasion of breast cancer cells *in vitro*. Meanwhile, Anxa2 depletion significantly ablated pulmonary metastasis in a severe combined immunodeficiency mouse model of breast cancer. Importantly, Anxa2 reduction inhibited EGF-induced activation of STAT3, which is required for EGF-induced EMT. Anxa2 directly bound to STAT3 and enhanced its transcriptional activity, thereby indicating that Anxa2 promotes EGF-induced EMT in a STAT3-dependent manner. Our findings provide clinical evidence that Anxa2 is a poor prognostic factor for breast cancer and reveal a novel mechanism through which Anxa2 promotes breast cancer metastasis.

## INTRODUCTION

Tumor metastasis accounts for approximately 90% of cancer-associated mortality [1]. In the past two decades, the mechanism of tumor metastasis is illustrated by the cell biological program epithelial–mesenchymal transition (EMT) [1-8]. During EMT, stationary epithelial cells lose polarity with cell-cell junctions and acquire a mesenchymal morphology and the ability to migrate and invade [3, 9]. EMT is an orchestrated series of events triggered by various inducers through numerous signal pathways [2, 3, 9]. The activation of the epidermal growth factor receptor (EGFR) signaling induces EMT in several types of carcinomas, including breast cancer [3, 10-16]. EGFR overexpression is also correlated with aggressive pathological features and poor outcome of breast cancer patients [17-19]. However, the molecular mechanisms underlying EGFR signaling-triggered EMT and cancer progression remains largely unclear and must be further investigated.

Annexin A2 (Anxa2), a calcium-dependent phospholipid-binding protein, is well-characterized as a receptor for plasminogen activator [20-22]. Anxa2 is involved in various cellular activities, including cell proliferation, adhesion, migration, invasion, and angiogenesis [23, 24]. Overexpression of Anxa2 was observed in many types of tumor tissues and was found to contribute to cancer progression [23, 24]. We previously showed that upregulated Anxa2 expression promotes the invasion of breast cancer cells, whereas Anxa2 knockdown inhibits cells invasive potential [25, 26]. Recently, the function of Anxa2 in EMT was reported in some studies. Upregulated Anxa2 expression is correlated with the expression of EMT markers in human adenomyosis [27]. Anxa2 is also required for TGF β-induced EMT in pancreatic ductal adenocarcinoma [28]. However, whether Anxa2 is involved in EMT induction in breast cancer or other types of tumors remains unknown. Thus, the detailed mechanism through which Anxa2 promotes EMT and cancer progression must be identified.

Anxa2 is a well-known substrate of the nonreceptor tyrosine kinase Src [29], which acts downstream of EGFR signaling [30, 31]. Interestingly, recent studies provided evidences that Anxa2 is involved in EGFR signaling [3, 16, 32], and blocking AnxA2 function or knockdown of Anxa2 expression inhibits the activation of the EGFR downstream pathway and reduces cell migration in breast cancer cell line MDA-MB-231 [33]. Given the important function of EGFR signaling in EMT induction in breast cancer, we speculate that Anxa2 promotes EMT via the activation of the EGF/EGFR pathway. In this study, the expression pattern of Anxa2 in breast cancer tissues was investigated. A strong positive correlation was found between Anxa2 and the expression of EGFR and EMT makers. Knockdown of Anxa2 also impaired EGF-induced EMT, as well as breast cancer cell invasion *in vitro* and metastatic potential *in vivo*. We further demonstrated that Anxa2 reduction inhibited EGF-induced activation of STAT3, which is required for EGF-induced EMT. We also provide evidence that Anxa2 directly bound to STAT3 and enhanced its transcriptional activity. Given that STAT3, a key EMT inducer, upregulated the expression of master EMT regulators, including Snail, Slug, and Twist [14, 34, 35]. Our findings suggest a novel mechanism through which Anxa2 promotes breast cancer EMT and metastasis.

## RESULTS

### Elevated expression of Anxa2 is positively correlated with breast cancer metastasis and EMT markers

**Figure 1:**
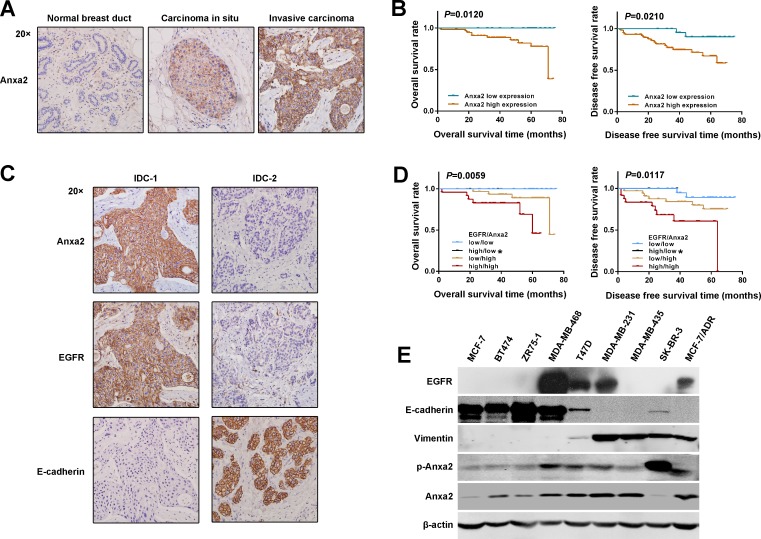
Elevated expression of Anxa2 is positively correlated with breast cancer metastasis and EMT markers **A.** Anxa2 staining intensity increased along with the tumor aggressiveness, as detected by IHC. Anxa2 expression was negative in normal breast ducts, moderate positive in DCIS, and strong positive in invasive cancer. **B.** The Kaplan-Meier method was used for survival analysis. The OS and DFS rates in patients with elevated expression of Anxa2 were significantly worse than those in patients with low expression of Anxa2 (*P* < 0.05). **C.** Expression of Anxa2 negatively correlated with E-cadherin expression and positively correlated with EGFR expression in breast cancer tissues. Anxa2, EGFR, and E-cadherin expression explored by IHC in two representative cases of invasive ductal breast cancer (IDC): E-cadherin negatively expressed in IDC-1 in which Anxa2 and EGFR were strongly expressed; IDC-2 was synchronously negative in Anxa2 and EGFR expression but had high E-cadherin expression. **D.** The OS and DFS rates in breast cancer patients with synchronously high expression of Anxa2 and EGFR were significantly worse than those in patients with synchronously low expression of Anxa2 and EGFR or those in patients with only Anxa2 upregulation (*P* < 0.05). *Only two cases in the Anxa2 low expression and EGFR high expression group survived without cancer relapse in our study. Survival analysis was performed using the Kaplan-Meier method. **E.** The expression of Anxa2, EGFR and EMT markers in a panel of breast cancer cell lines were analyzed by Western blotting.

Anxa2 overexpression has been reported in many malignant tumors. However, the data in breast cancer remain limited. In our previous study [36], we showed that elevated expression of Anxa2 mRNA correlates with lymph node metastasis of invasive ductal carcinoma (IDC). Here, we investigated the relationship between Anxa2 protein expression and breast cancer prognosis by immunohistochemistry. We observed that Anxa2 expression was negative in normal breast ducts, moderately positive in ductal carcinoma in situ, and strongly positive in most of the invasive cancer (76/85) (Figure 1A). The increase in staining intensity along with the tumor aggressiveness indicates a possible promoting role of Anxa2 in breast tumorigenesis. Clinical and pathological parameter analysis revealed that elevation expression of Anxa2 significantly correlated with lymph node invasion, advanced pathological stage, and higher histological grade (Table 1, *P <* 0.05). Moreover, in good consistence with the worse histopathological features, survival analysis showed significantly worse overall survival (OS) (*P* = 0.0120) and worse disease free survival (DFS) (*P* = 0.0210) in Anxa2 high expression group (Figure 1B). The above results suggested an association between high expression of Anxa2 and breast cancer progression.

**Table 1:** Correlation of Anxa2 expression with cliniclpathological parameters

Parameters	No of Anxa2 expression (%)		*P* value
	Low	High	
Age group			0.1508
≤40	4 (16.7%)	4 (6.6%)	
>40	20 (83.3%)	57 (93.4%)	
Menopausal status			0.4020
Premenopausal	9 (37.5%)	29 (47.5)	
Postmenopausal	15 (62.5%)	32 (52.5)	
Tumor diameter			0.1110
≤5cm	24 (100.0%)	55 (90.2%)	
>5cm	0 (0.0%)	6 (9.8%)	
Lymphnode invasion			<0.0001*
Negative	21 (87.5%)	24 (39.3%)	
Positive	3 (12.5%)	37 (60.7%)	
Pathological stage			0.0003*
I,II a	21 (87.5%)	27 (44.3%)	
II b, III	3 (12.5%)	34 (55.7%)	
Histological grade			0.0083*
I, II	24 (100.0%)	47 (77.0%)	
III	0 (0.0%)	14 (23.0%)	
ER status			0.4819
Negative	9 (37.5%)	28 (45.9%)	
Positive	15 (62.5%)	33 (54.1%)	
PR status			0.0307*
Negative	6 (25.0%)	31 (50.8%)	
Positive	18 (75.0%)	30 (49.2%)	
HER2 status			0.2782
Negative	21 (87.5%)	47 (77.0%)	
Positive	3 (12.5%)	14 (23.0%)	

To assess the relationship between Anxa2 overexpression and EMT signature, we detected the epithelial marker E-cadherin expression given that the loss of E-cadherin is a fundamental event in EMT. We observed a significant low expression of E-cadherin in the Anxa2 high expression group (Figure 1C and Table 2, *P* = 0.0001), supporting a functional association between Anxa2 overexpression and breast cancer EMT development. Whether Anxa2 serves a function in EGFR signaling and promotes EMT has attracted our interest, then we tried to seek evidence in human tissue specimens. As shown in Figure 1C and Table 2, EGFR was highly expressed in the Anxa2 high expression group than in Anxa2 low expression group (*P* = 0.0021). Interestingly, in both EGFR and Anxa2 high expression groups, E-cadherin presented a significantly higher rate of low expression (Table 2, *P* = 0.0002), which indicates a combined effect of EGFR and Anxa2 on breast cancer EMT. As expected, the effect on EMT might induce the worst outcome in EGFR/Anxa2 coinstantaneous high expression group, as revealed by the survival analysis (Figure 1D, *P* < 0.05).

Subsequently, a panel of human breast cancer cell lines was screened for Anxa2, EMT markers, and EGFR expression by Western blotting analysis. As shown in Figure 1E, Anxa2 was highly expressed in all the EGFR positive breast cancer cell lines, and strongly positive expression of Anxa2 was found in cell lines that were characterized as mesenchymal-like and highly aggressive, such as MDA-MB-231, MDA-MB-435 and MCF-7/ADR. In mesenchymal-like SK-BR-3 cells, Anxa2 was expressed at a low level, but the expression level of its tyrosine phosphorylation was significantly increased, which plays a critical role in cancer cells EMT and metastasis [28, 29]. Taken together, these results strongly indicate that elevated expression of Anxa2 and EGFR has a direct association with EMT in breast cancer.

**Table 2:** Correlation of Anxa2 expression with E-cadherin and EGFR expression

	No of Anxa2 expression (%)		*P* value
	low	high	
EGFR			0.0021*
low	22 (91.7%)	35 (57.4%)	
high	2 (8.3%)	26 (42.6%)	
E-cadherin			0.0001*
low	1 (4.2%)	28 (45.9%)	
high	23 (95.8%)	33 (54.1%)	
	No of E-cadherin expression (%)		*P* value
	low	high	
EGFR/Anxa2			0.0002*
low/low	1 (4.5%)	21 (95.5%)	
low/high, high/low	12 (32.4%)	25 (67.6%)	
high/high	16 (61.5%)	10 (38.5%)	

### EGF-induced EMT is inhibited by Anxa2 knockdown and depends on 23 tyrosine phosphorylation of Anxa2

To clarify the effect of Anxa2 on EMT and EGFR signaling, two EGFR-positive and epithelial-like breast cancer cell lines T47D and MDA-MB-468 were used to establish EGF-induced EMT switch models. Exposure to exogenous EGF for 72 h induced an EMT-like morphological change in both cell lines, whereby cells lost their cell-cell junction and became elongated and scattered in comparison with control group (Figure S1A). In addition, EGF also led to a significantly loss of epithelial marker E-cadherin and a slight increase of Vimentin in T47D cells, which further support an EMT of T47D cells (Figure S1B). In MDA-MB-468 cells, EGF induced a significantly upregulation of mesenchymal marker Vimentin, however, the change in E-cadherin expression could barely be observed. Given that Snail, Slug, and Twist are the well-established EMT drivers [3], we examined the mRNA expression of the three transcription factors by RT-PCR and found an upregulation in Slug rather than Snail and Twist in both cell lines after EGF exposure (data not shown). Consistently, elevated Slug expression after EGF exposure in the two cell lines were observed using Western blotting analysis (Figure S1B). Moreover, immunofluorescence staining assay showed an elevated expression of Vimentin as well as Slug in these two cell lines after EGF treatment (Figure S1C). EGF also induced a significantly reduction of E-cadherin in T47D cells; interestingly, although total E-cadherin protein in MDA-MB-468 cells exhibited no obvious change in Western blotting analysis, immunostaining assay showed that its expression in the membrane and cell-cell junction was reduced, thereby indicating its functional loss. Taken together, these data demonstrate that the EMT model induced by exogenous EGF was successfully established.

**Figure 2:**
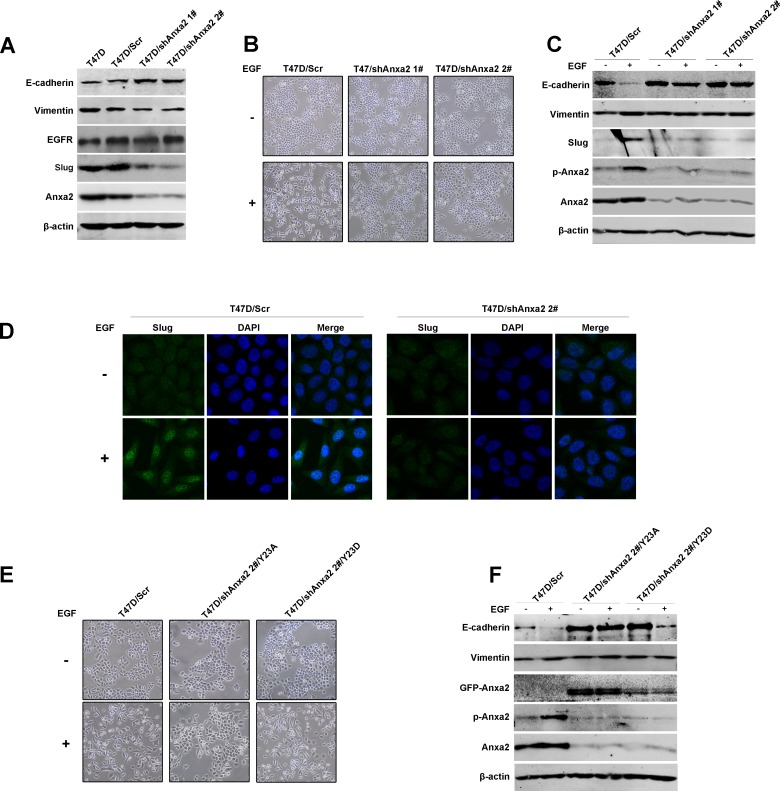
Anxa2 is required for EGF-induced EMT in human breast cancer cells **A.** A mesenchymal-epithelial transition (MET)-like pattern was observed after Anxa2 knockdown in human breast cancer T47D cells. Western blotting analysis of expression of Anxa2, EGFR and EMT markers in T47D cells, and T47D cells infected with lentivirus expressing control or two Anxa2 specific shRNAs. **B.** Depletion of Anxa2 expression inhibited EGF-induced EMT morphological switch. Control and Anxa2 knockdown T47D cells were serum-starved for 24 h, then the cells were cultured in 0.5% FBS medium with or without EGF for 72h. The cell morphological changes were visualized under a microscopy. **C.** Knockdown of Anxa2 inhibited EGF induced EMT in T47D cells. Control and Anxa2 knockdown T47D cells were serum-starved for 24 h, treated with or without EGF for 72 h, then the expression of Anxa2, phosphorylated Anxa2 and EMT markers were analyzed by Western blotting method. **D.** Confocal immunoﬂuorescence microscopy analysis showed that Anxa2 knockdown suppressed EGF-induced Slug accumulation in nucleus. **E.** Tyrosine phosphorylation of Anxa2 is required for EGF induced cell morphological change in T47D breast cancer cells. The Anxa2-depleting T47D cells were stable transfected with plasmids expressing either the phospho-mimicking mutant form of AnxA2 (Anxa2^Y23D^) or the phosphorylation-deficient mutant form of Anxa2 (Anxa2^Y23A^), then the cells were treated with or without EGF for 72h. The cell morphological changes were visualized under a microscopy. **F.** Re-expression of Anxa2^Y23D^ rescued EGF induced EMT in Anxa2 knockdown T47D cells. Western blotting analysis of expression of EMT markers in control, and Anxa2 knockdown T47D cells transfected with Anxa2^Y23D^ or Anxa2^Y23A^ plasmids.

To verify whether Anxa2 is crucial for EMT, Anxa2 was stable knockdown in T47D cells using lentivirus mediated shRNAs. As shown in Figure 2A, a mesenchymal-epithelial transition (MET)-like pattern was observed after Anxa2 knockdown in T47D cells, as evidenced by the upregulation of epithelial marker E-cadherin expression, downregulation of mesenchymal marker Vimentin, as well as Slug expression (Figure 2A). However, EGFR expression was unaffected in Anxa2 knockdown cells. The control and Anxa2-depleting cells were then treated with chronic EGF for 72 h. As shown in Figure 2B, the morphological switch from epithelial-like phenotype to mesenchymal-like phenotype induced by EGF was blocked in shAnxa2 cells. Likewise, Anxa2 depletion also inhibited EGF induced expression changes of EMT associated markers in comparison with that of in control cells (Figures 2C and 2D). Overall, our data support the critical function of Anxa2 in EMT development induced by EGF.

Phosphorylation at tyrosine 23 plays a crucial role for Anxa2 in promoting cancer cell migration, invasion and metastasis [29]. In this study, we observed that EGF induces EMT of breast cancer cells along with constantly tyrosine phosphorylation of Anxa2 (Figure S1B). To investigate whether phosphorylation of Anxa2 at Tyr23 contributes to EGF induced EMT, we constructed a panel of plasmids expressing either phospho-mimicking mutant form of AnxA2 (Anxa2^Y23D^), in which Tyr 23 was mutated to an aspartic acid, or the phosphorylation-deficient mutant form of Anxa2 (Anxa2^Y23A^), in which Tyr23 was mutated to an alanine. Then plasmids expressing Anxa2 mutants were transfected into Anxa2-depleting cells T47D/shAnxa2 2#, in which the cells expressing a shRNA targeting the non-coding region of Anxa2 mRNA. As shown in Figure 2E and 2F, re-expression of Anxa2^Y23D^, but not Anxa2^Y23A^, in Anxa2-depleting cells recued EGF induced EMT. These results suggested that tyrosine phosphorylation of Anxa2 is required for EGF induced EMT in breast cancer cells.

### Depletion of Anxa2 expression impairs invasive potential of T47D cells *in vitro* and *in vivo*

**Figure 3:**
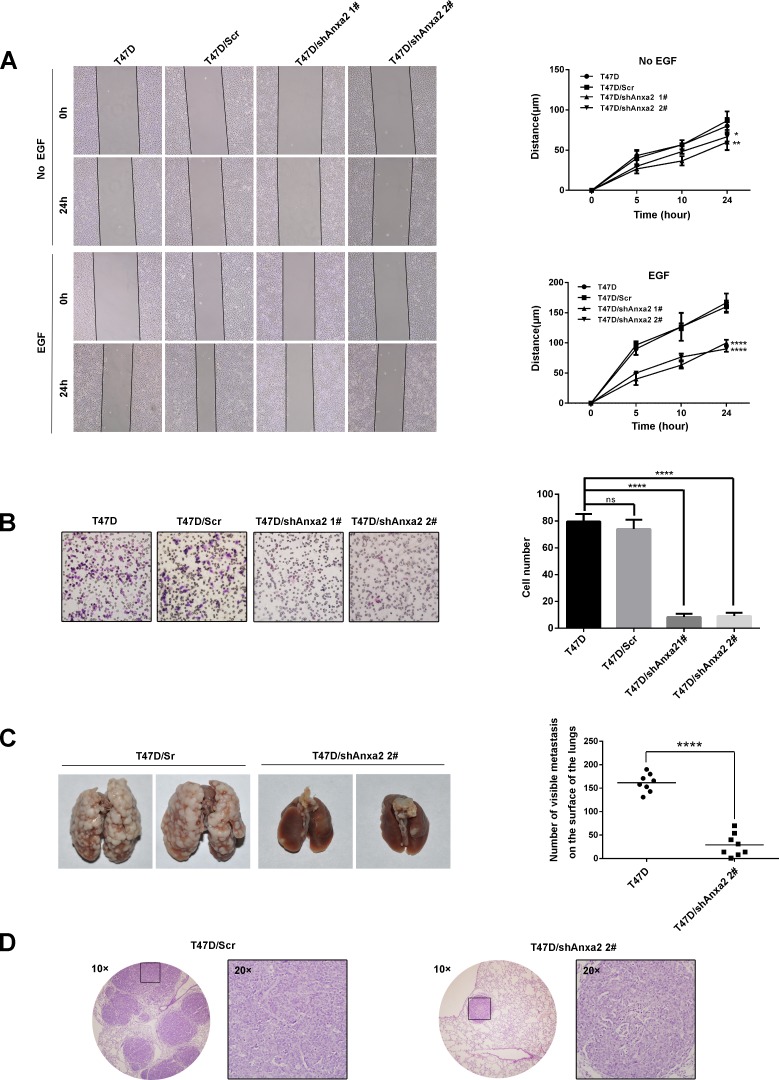
Depletion of Anxa2 expression impairs invasive potential of T47D cells *in vitro* and *in vivo* **A.** Knockdown of Anxa2 inhibited cell migration ability in the presence or absence of EGF. Wound healing assay of T47D and T47D cells infected with control or shAnxa2-expressing lentivirus. Relative cell migration distance was quantiﬁed and plotted in the right panel. Data as mean ± SD of triplicates, *P* < 0.05. **B.** Knockdown of Anxa2 expression inhibited cell invasion ability of T47D cells. Transwell based assay was conducted to analyze the cell invasion capability. 10 ng/mL of EGF was added into the lower chamber as the chemotactic factor. After 24h, invaded cells were counted in five randomly chosen areas under a microscopy, and the average of triplicate experiments was summarized in the right panel. Data as mean ± SD, *P* < 0.0001. **C.** Anxa2 depletion significantly inhibited pulmonary metastasis in a severe combined immunodeficiency (SCID) mouse model of breast cancer. Anxa2 silencing (shAnxa2 2#) and control cells were injected into caudal veins of SCID mice. After six weeks, the mice were sacrificed by anesthetic overdose. The lung tissues were anatomized, and the number of lung metastases was counted and plotted in the right panel (*P* < 0.0001). **D.** Human tumor foci on mouse lungs were confirmed by H & E staining, and visualized under a light microscopy. Statistical analysis of cell migration capability was performed by a two-way ANOVA followed by Dunnett's multiple comparison test. Statistical analysis of cell invasion capability was performed by a one-way ANOVA followed by Dunnett's multiple comparison test. Numbers of metastasis *in vivo* was statistically analyzed by unpaired t test. ns: no significance; * *P* < 0.05; **P < 0.01; ****P < 0.0001.

EMT is critical for cancer cells migration and invasion, we next investigate the effect of Anxa2 knockdown on breast cancer cell migration ability, as shown in Figure 3A, silence of Anxa2 significantly decreased wound healing induced cell migration in the presence or absence of EGF (*P* < 0.05). EGF could induce EMT switch, and we observed subsequent increase of cell migration after EGF treatment. However, since EMT was inhibited by Anxa2 knockdown, shAnxa2 cells were significantly less sensitive toward EGF-enhanced cell migration comparing to wild type and control T47D cells. Consistently, Anxa2 knockdown cells also showed attenuated invasion ability as measured in a transwell based assay using EGF as the chemotactic factor (Figure 3B, *P* < 0.0001).

To investigate the effect of Anxa2 knockdown on the metastatic potential of T47D cells *in vivo*, Anxa2 silencing cells and control cells were injected into the caudal veins of 5-6-week-old SCID mice. The mice were euthanized using an overdose of anesthesia after six weeks when obvious consumption status occurred. Lung tissues were anatomized and fixed in 4% paraformaldehyde. The numbers of lung metastases in Anxa2 depletion cells group were significantly reduced in comparison with that of in control group. (Figure 3C, *P* < 0.0001). Moreover, results from HE staing also showed that the volume of metastatic foci seemed smaller in animals injected with shAnxa2 cells than in control cells (Figure 3D). These data provided further evidence that Anxa2 is critical for the metastatic potential of breast cancer cells *in vivo*.

### Anxa2 is essential for EGF-induced STAT3 activation

**Figure 4:**
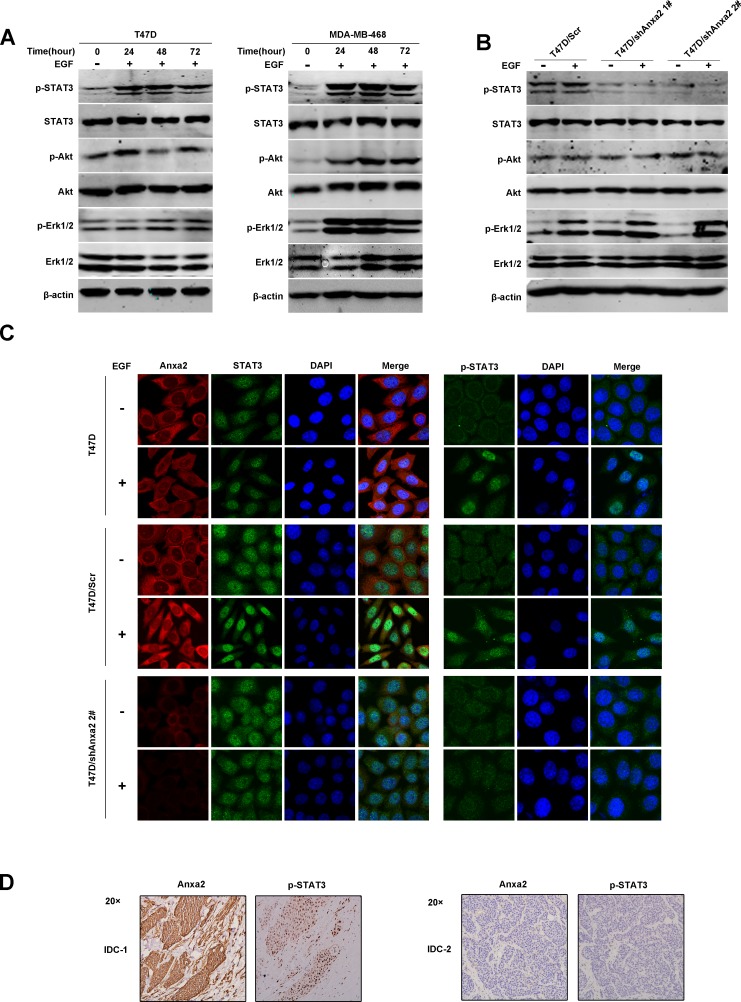
Anxa2 reduction decreases EGF-induced STAT3 activation **A.** Western blotting analysis of EGFR downstream signaling after EGF stimulation in T47D and MDA-MB-468 cells. These two breast cancer cell lines were grown to 80%-90% confluence, starved for 12 hours, and stimulated with EGF for 0, 24, 48, and 72 h, then total cellular protein were extracted and subjected to Western blotting analysis. **B.** Western blotting analysis indicated that the phosphorylation of STAT3 at Tyr 705 triggered by EGF was blocked by Anxa2 depletion in T47D cells. **C.** Confocal immunoﬂuorescence microscopy analysis showed that Anxa2 knockdown inhibited EGF induced nuclear translocation of STAT3 in T47D cells. Wild type, control and Anxa2 knockdown T47D cells were starved for 12 hours, and stimulated with or without EGF for 12 h, then the subcellular distribution of Anxa2, STAT3, and tyrosine phosphorylated STAT3 were detected using immunofluorescence staining method. **D.** Elevated expression of phospho-STAT3 (Tyr705) was found in breast cancer tissues with Anxa2 high expression. A significant positive correlation between Anxa2 and phosphorylated STAT3 expression was observed in human breast cancer tissues (*P* < 0.05).

To further investigate the mechanism by which Anxa2 affects EGF-induced EMT, we examined the activation of EGFR downstream signaling pathway in T47D and MDA-MB-468 cells. As shown in Figure 4A, STAT3, Erk1/2 and Akt phosphorylation were obviously increased during the EMT procedure, indicating the activation of these signaling pathways. We then determine the effect of Anxa2 knockdown on EGF/EGFR signaling activation, as shown in Figure 4B, silencing of Anxa2 in T47D cells has no significant effect on activation of Erk1/2 and Akt, however, the phosphorylation of STAT3 at Tyrosine 705 was blocked (Figure 4B). STAT3 is well known to serve as a transcription factor whose tyrosine phosphorylation is critical for its dimerization, nuclear translocation, and thus activation [35]. We then explored the changes in the subcellular location of Anxa2 and STAT3 before and after EGF stimulation using immunofluorescence staining. As expected, EGF induced significant nuclear translocation of STAT3 from cytoplasm in wild-type and control T47D cells, and its active form p705Tyr-STAT3 was obviously increased in the nucleus in parallel(Figure 4C). Meanwhile, we also detected a certain amount of Anxa2 translocated into the nucleus after EGF treatment (Figure 4C). However, the nuclear translocation of STAT3 and expression of Tyrosine phosphorylated STAT3 triggered by EGF was markedly decreased in Anxa2 reduction cells (Figure 4C). More interestingly, results from IHC analysis of human breast cancer tissues supported the contribution of Anxa2 to the activation of STAT3. Elevated expression of phospho-STAT3 (Tyr705) was found in the Anxa2 high expression group (Figure 4D and Table 3, *P* = 0.0154). Altogether, the data suggest that Anxa2 is required for STAT3 activation triggered by EGF.

**Table 3:** Correlation of Anxa2 expression with pTry705-STAT3 expression

	No of Anxa2 expression (%)		*P* value
	Low	high	
p-STAT3			0.0154*
low	22 (91.7%)	40 (65.6%)	
high	2 (8.3%)	21 (34.4%)	

### STAT3 is required for EGF-induced EMT in breast cancer cells

**Figure 5:**
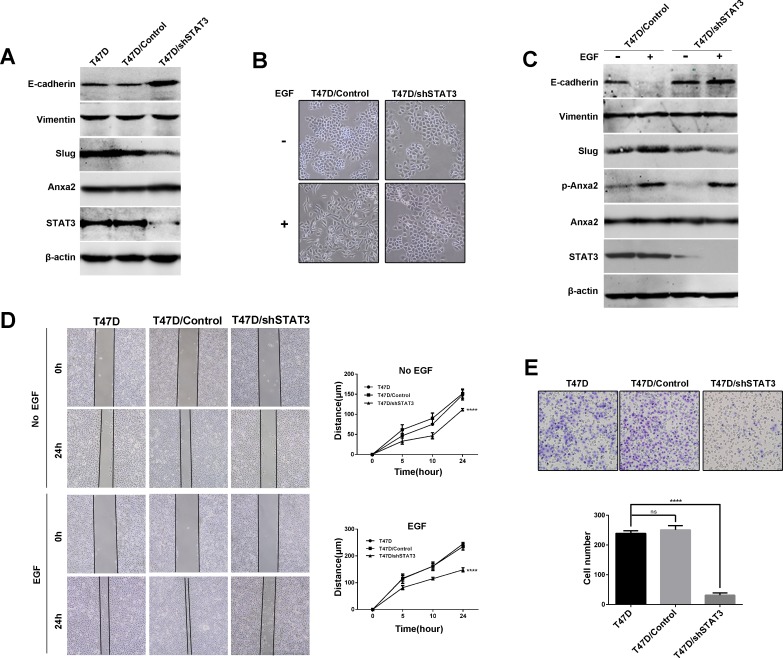
Downregulation of STAT3 expression inhibits EGF-induced EMT and cell migration and invasion in T47D cells **A.** Knockdown of STAT3 expression resulted in upregulation of E-cadherin and downregulation of Slug in T47D cells. **B.** STAT3 knockdown inhibited EGF induced cell morphological change in T47D cells. Control and STAT3 knockdown cells were serum-starved for 24 h, then the cells were cultured in 0.5% FBS medium with or without EGF for 72h, the morphological changes was visualized under a microscopy. **C.** STAT3 knockdown inhibited EGF induced downregulation of E-cadherin and upregulation of slug. Control and STAT3 knockdown T47D cells were starved, and treated with or without EGF for 72 h, then the expression of E-cadherin, Vimentin, slug, Anxa2 and phosphorylated Anxa2 were analyzed by Western blotting method. **D.** Knockdown of STAT3 inhibited cell migration ability in the presence or absence of EGF. Wound healing assay of T47D and T47D cells infected with control or shSTAT3-expressing lentivirus. Relative cell migration distance was quantiﬁed and plotted in the right panel. Data as mean ± SD of triplicates. Statistical analysis was performed by two-way ANOVA followed by Dunnett's test (*P* < 0.0001). **E.** Knockdown of STAT3 expression inhibited cell invasion ability of T47D cells. Data as mean ± SD, *P* < 0.0001. Statistical analysis was performed by one-way ANOVA followed by Dunnett's test.

Since STAT3 is a well-known EMT promoter in tumors [35], we hypothesized that STAT3 plays a key role in our EGF-regulated EMT model. We next knocked down STAT3 expression using lentivirus mediated shRNA. We observed upregulation of E-cadherin and downregulation of Slug after STAT3 depletion in T47D cells (Figure 5A). EGF was then used to trigger EMT. As shown in Figure 5B and 5C, EGF induced morphological changes and expression of EMT associated protein markers were significantly inhibited by STAT3 knockdown in comparison with the control cells. However, EGF induced phosphorylation of Anxa2 was not blocked in STAT3 knockdown cells, suggesting that STAT3 was a downstream factor of Anxa2 to affect EMT (Figure 5C). We also examined the effect of STAT3 knockdown on migration and invasion ability in T47D cells. As shown in Figure 5D, downregulation of STAT3 impaired wound healing induced cell migration in the presence or absence of EGF (*P* < 0.0001). Consistently, transwell assay also showed that the depletion of STAT3 also attenuated cell invasion in response to EGF (Figure 5E, *P* < 0.0001). Thereupon, we proved that STAT3 is a key factor in EGF-induced EMT. Altogether, our results demonstrated that Anxa2 mediated EMT induced by EGF through STAT3 pathway.

### Anxa2 has a direct interaction with STAT3

**Figure 6:**
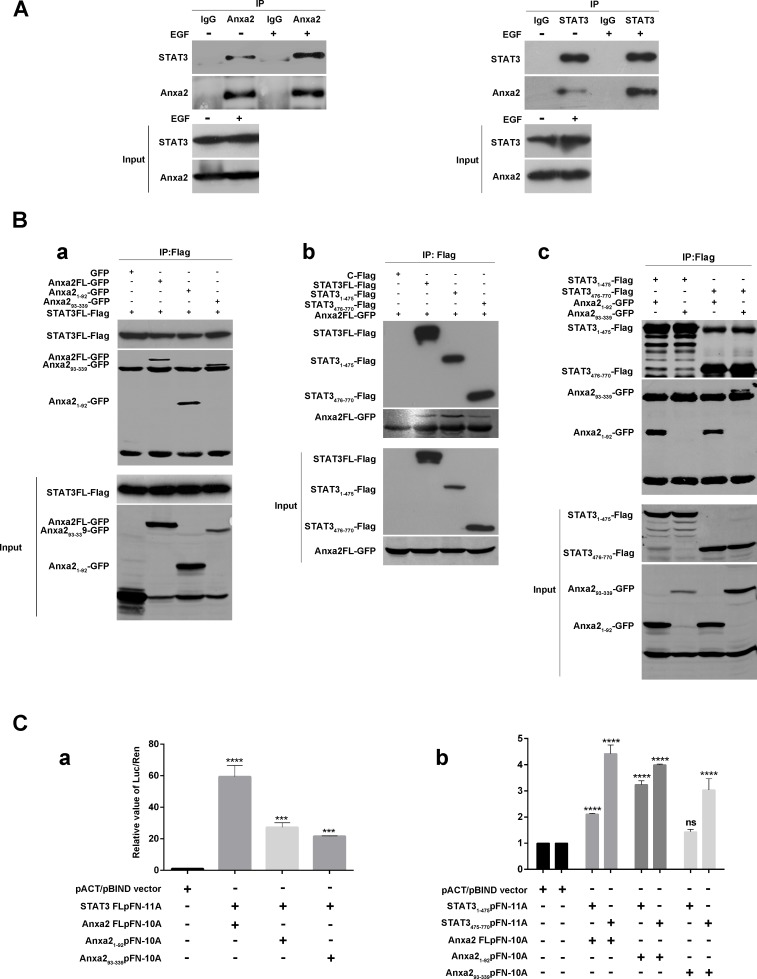
Anxa2 binds directly to STAT3 in breast cancer cells **A.** Co-immunoprecipitation assay showed that the interaction between endogenous STAT3 and Anxa2 was increased after EGF stimulation in physiological condition. The T47D cells were serum-starved for 24 h, then the cells were stimulated with or without EGF for 72h, and co-immunoprecipitation assays were conducted using anti-Anxa2 (left) or anti-STAT3 (right) antibodies. **B.** GFP-tagged Anxa2 binds to Flag-tagged STAT3 in HEK 293T cells. a: Full length STAT3 binds to both Anxa2 N-terminus fragment 1-92aa and its C-terminus fragment 93-339aa. HEK-293T cells were cotransfected with Flag-tagged STAT3 plasmid and different fragments of GFP-tagged Anxa2 expression vectors. After transfection of 48 hours, the cells were lysed, co-immunoprecipitated with anti-Flag antibodies conjugated beads, and analyzed by western blotting with anti-Flag or GFP antibody. b: GFP- tagged full length Anxa2 binds to both STAT3 N-terminus fragment 1-475aa and its C-terminus fragment 476-770aa. HEK-293T cells were cotransfected with a GFP-tagged full length Anxa2 plasmid and different fragments of Flag-tagged STAT3 plasmids. Then the cell lysates were co-immunoprecipitated with anti-Flag antibodies conjugated beads, and analyzed by western blotting with anti-Flag or GFP antibody. c: N-terminal region of Anxa2 binds to both the N- and C- terminal domains of STAT3, whereas its C- terminal fragment containing 93-339aa only binds to C- terminal domain of STAT3. **C.** Anxa2 directly binds to STAT3. CheckMate™/Flex^i®^ Vector Mammalian Two-Hybrid System was used to determine the direct protein-protein interaction between Anxa2 and STAT3. a: pFN11A vectors containing full length STAT3, and pFN10A vectors containing full length or different fragments of Anxa2 were co-transfected into 293T cells and cultured for 48h. Then the cells were lysed, and the relative value of Luc/Ren was tested. b: pFN11A vectors containing different fragments of STAT3, and pFN10A vectors containing full length or different fragments of Anxa2 were co-transfected into 293T cells and cultured for 48h. Then the cells were lysed, and the relative value of Luc/Ren was tested. Data as mean ± SD of triplicates. Statistical analysis was performed by One-way ANOVA followed by Dunnett's test. ns: no significance; ***P < 0.001; ****P < 0.0001.

Given the fact that Anxa2 mediated STAT3 phosphorylation, and that EGF induced nuclear translocation of both STAT3 and Anxa2 (Figures 4B and 4C), we hypothesized that Anxa2 may be a binding partner of STAT3. Thus, we investigated the possibility that Anxa2 interacts with STAT3 in T47D cells treated with or without EGF using co-immunoprecipitation assay. As shown in Figure 6A, endogenous Anxa2 coprecipitated with endogenous STAT3 in the absence of EGF stimulation, and this interaction was enhanced on EGF stimulation. In addition, reciprocal co-immunoprecipitation assay using anti-STAT3 antibodies also confirmed that EGF could increase the interaction between STAT3 and Anxa2 in physiological condition (Figure 6A). These results suggest that Anxa2 was a novel binding protein of STAT3. To further determine how Anxa2 interacts with STAT3, two sets of plasmids expressing different length of Anxa2 or STAT3 proteins were constructed. Then these plasmids were transfected into HEK293T cells and co-immunoprecipitation assay were performed. In 293T cells cotransfected with a Flag-tagged full length STAT3 expression vector and different fragments of GFP-tagged Anxa2 expression vectors, STAT3 was coprecipitated with both Anxa2 N-terminus fragment 1-92aa and its C-terminus fragment 93-339aa (Figure 6Ba). Moreover, in 293T cells cotransfected with a GFP-tagged full length Anxa2 plasmid and different fragments of Flag-tagged STAT3 plasmids, Anxa2 also coprecipitated with both STAT3 N-terminus fragment 1-475aa and its C-terminus fragment 476-770aa (Figure 6Bb). Further mapping analysis showed that the N-terminal region of Anxa2 could interact with both the N- and C- terminal domains of STAT3, whereas its C- terminal fragment containing 93-339aa could only interact with C- terminal domain of STAT3 (Figure 6Bc).

Next, in order to determine whether there is a direct interaction between Anxa2 and STAT3, *in vitro* protein-protein interaction assay were performed using the CheckMate™/Flexi^®^ Vector Mammalian Two-Hybrid System. Full length or different fragments of STAT3 were cloned into pFN11A (BIND) vectors, and full length or different fragments of Anxa2 were cloned into pFN10A (ACT) vectors, then pFN11A, pFN10A (ACT) and pGL4.31vectors were co-transfected into 293T cells. The interaction between Anxa2 and STAT3 was determined by measuring the relative luciferase activity. Finally, we confirmed the existence of a direct interaction between Anxa2 and STAT3 (Figure 6Ca). In agreement with the results of co-immunoprecipitation assays (Figure 6Cb), the relative luciferase activity from the co-transfection of N-terminal fragment of Anxa2 and N- or C-terminal domain of STAT3 was significantly higher than that of negative control groups. Similar results was also obtained from the co-transfection of C-terminal fragment of Anxa2 and C-terminal domain of STAT3 group, but not from that of co-transfection of C-terminal fragment of Anxa2 and N-terminal domain of STAT3 group. Moreover, Anxa2 and its two fragments had stronger interaction with C-terminal domain of STAT3 than the N-terminal fragment, and the N-terminal fragment of Anxa2 had stronger interaction with C- terminal domain of STAT3 than the C-terminal fragment of Anxa2. Since C- terminal domain of STAT3 contains the phosphorylation site Try705 and the transactivation domain, and N-terminal fragment of Anxa2 contains the phosphorylation site Try23, we speculate that phosphorylation of Anxa2 may regulate the phosphorylation and transcriptional activity of STAT3 through the direct interaction. Altogether, our results indicated a direct interaction between Anxa2 and STAT3 in breast cancer cells.

### Anxa2 regulates the transcriptional activity of STAT3

**Figure 7:**
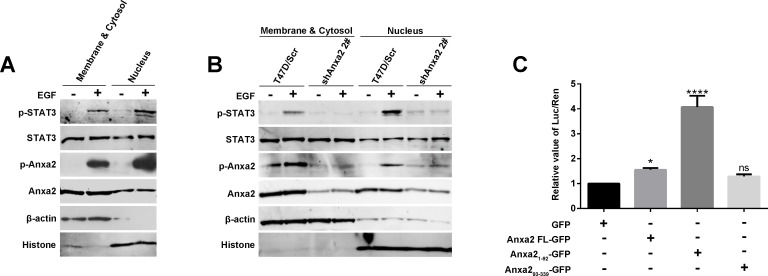
Anxa2 enhances the transcriptional activity of STAT3 **A.** The nuclear distribution of phosphorylated STAT3 and phosphorylated Anxa2 was increased after EGF stimulation in T47D cells. The T47D cells were serum-starved for 24 h, then the cells were treated with or without EGF for 72h. The cytosolic and nuclear fractions were prepared, and analyzed by western blotting method. β-actin as loading control for cytosolic fraction, and histone as loading control for nuclear fraction. **B.** EGF-induced nuclear distribution of phosphorylated STAT3 was markedly reduced in Anxa2 knockdown T47D cells than in control cells. **C.** Dual-luciferase reporter assay showed that full-length Anxa2 and Anxa2 N-terminus fragment significantly enhanced the transcriptional activity of STAT3. A Stat3-specific reporter plasmid and full length or different fragments of Anxa2 vectors were cotransfected into HEK 293T cells. After transfection for 48 h, the cells were lysed, and the relative value of Luc/Ren was tested. Data as mean ± SD of triplicates. Statistical analysis was performed by One-way ANOVA followed by Dunnett's test. ns: no significance; * *P <* 0.05; ****P < 0.0001.

STAT3 is an important transcription factor for EMT [35]. Since Anxa2 has a strong interaction with the STAT3_476-770_ fragment containing transactivation domain, we assume that Anxa2 may regulate STAT3 transcriptional activity. As shown in Figure 4, Anxa2 mediated phosphorylated activation and translocation into the nucleus of STAT3, and a certain amount of nuclear Anxa2 was found. We expected a spatial interaction between Anxa2 and STAT3, which facilitates the regulative effect of Anxa2 on transcriptional activity. We separated nuclear protein from cytoplasmic and membranous protein fractions for Western blotting analysis to verify the spatial interaction. Expression of pTyr705-STAT3 remarkably increased, especially in the nucleus simulated by EGF, and pTyr23-Anxa2 expression likewise increased in the nucleus (Figure 7A). To further confirm the effect of Anxa2 activation on STAT3 spatial translocation, we examined the nuclear expression of STAT3 and p705Tyr-STAT3 in Anxa2 knockdown cells and control cells. Nuclear translocation of STAT3 under the effect of EGF was cut down in shAnxa2 cells (Figure 7B).

A dual-luciferase reporter assay system was used to study the effect of Anxa2 on STAT3 transcriptional activity. Compared with the case of the control group, full-length Anxa2 and the N-terminal fragment enhanced the transcriptional activity of STAT3 (Figure 7C). Interestingly, the N-terminal fragment of Anxa2 containing Tyr 23 phosphorylation site had the strongest enhancive effect, which corresponded with the coincident special location of pTyr23-Anxa2 and pY705-STAT3 after EGF stimulation. These data suggest the important function of Tyr 23 phosphorylation of Anxa2 in the regulation of STAT3 transcriptional activity. In conclusion, we suggest that Anxa2 directly interacts with STAT3, and its phosphorylated activation modulates STAT3 phosphorylation at 705 tyrosine and translation into the nucleus to serve its function as a transcriptional factor for upregulating Slug.

## DISCUSSION

Anxa2 is a hotspot molecule with potential to be a diagnostic and prognostic marker for certain cancers [23, 24]. In human breast cancer tissues, Anxa2 was found to be selectively expressed in IDC and DCIS but undetected in normal ductal epithelium and hyperplastic tissues [37]. However, no clinical or prognostic data were reported. In this study, we observed that Anxa2 expression was upregulated in aggressive type of breast cancer tissues, similar to the findings of previous studies. Overexpression of Anxa2 was frequently detected in patients with poor pathological features and outcomes. We proved for the first time that the upregulated Anxa2 expression is correlated with cancer metastasis and poor outcomes in breast cancer patients. A strong positive correlation was found between Anxa2 and EGFR overexpression in breast cancer tissues. Patients with high EGFR and Anxa2 expression showed significantly poor overall survival and disease-free survival rates. EGFR overexpression was observed in most clinically aggressive subtypes of breast cancer, including triple-negative breast cancer and inflammatory breast cancer [18, 38], and predicted poor prognosis in cancer patients [19]. Thus, our findings suggest a possible functional correlation between Anxa2 and EGFR during breast cancer aggravation.

EMT, a pivotal cellular process that promotes metastasis initiation, is induced by the activation of EGFR signaling in breast cancer [10, 11, 13]. Several studies revealed that Anxa2 is involved in EMT in avian heart development and adenomyosis [27, 39]. In this study, upregulated Anxa2 expression was significantly negatively correlation with low E-cadherin expression in breast cancer tissues and cell lines. Moreover, knockdown of Anxa2 expression resulted in a more epithelial phenotype in T47D cells. These data indicate the probable involvement of Anxa2 in EMT during cancer progression. This hypothesis was confirmed by the present results, which showed that Anxa2 knockdown inhibited EGF-induced cell morphological switch and altered the expression of EMT-associated markers. Similarly, Anxa2 depletion reduced breast cancer migration and invasion *in vitro* and metastatic potential of T47D cells in an SCID mouse model *in vivo*. A previous report showed that tyrosine phosphorylation of Anxa2 is required for TGF β-induced EMT in PDA [28]. Consistently, in the present study, re-expression of the phosphor-mimicking mutant Anxa2 (Anxa2 Y23D), but not the phosphorylation-deficient Anxa2 (Anxa2 Y23A), in Anxa2-silencing breast cancer cells rescued EGF-induced EMT. This observation further supported the hypothesis that Anxa2 is required for EGF-induced EMT in breast cancer cells.

Recent studies have reported that Anxa2 is involved in EGFR signaling [3, 16, 32]. Nevertheless, the mechanism through which Anxa2 promotes EGF-induced EMT in breast cancer cells remains unknown. In our EMT model, the phosphorylation of Akt, Erk1/2, and STAT3 was evidently enhanced, thereby indicating that EGFR signaling was activated during EMT. Meanwhile, the phosphorylation of Akt and Erk1/2 was not significantly altered in Anxa2-silencing cells, whereas EGF-induced STAT3 phosphorylation was significantly reduced compared with that in control cells. Consistently, EGF-induced nuclear translocation of STAT3 was also blocked. In human breast cancer tissues, phospho-STAT3 expression was upregulated in the Anxa2 high expression group. Together, these evidences suggest that Anxa2 is required for EGF-induced activation of STAT3 in breast cancer cells. Given that STAT3 is a well-known key pathway downstream of EGFR and an EMT promoter in breast cancer [35], we infer that the possible mechanism through which Anxa2 promotes EMT is dependent on the STAT3 pathway. In agreement with this hypothesis, our results showed that depletion of STAT3 expression in T47D cells significantly upregulated E-cadherin expression but downregulated Slug expression. Moreover, EGF-induced EMT was inhibited in STAT3-silencing T47D cells. Overall, these results suggest a novel mechanism through which Anxa2 promotes EGF-induced EMT in a STAT3-dependent manner.

The activation of STAT3 requires tyrosine phosphorylation by the upstream tyrosine kinases and subsequent translocation from the cytosol to the nucleus [34, 40-42]. In addition to tyrosine kinases, the formation of multiprotein “transducisome” with other proteins is considered critical for STAT3 activation. These proteins may serve as scaffold or adaptor proteins to promote the binding of STAT3 to the upstream kinases or downstream targets [43-45]. In this study, an interestingly ﬁnding is that EGF induced STAT3 nuclear translocation, along with Anxa2 upregulation in the nucleus. EGF also enhanced the co-immunoprecipitation between Anxa2 and STAT3 under physiological condition. Reciprocal co-immunoprecipitation assays using exogenous Anxa2 or STAT3 fragments confirmed that Anxa2 is a novel binding protein of STAT3. The CheckMate™/Flexi^®^ Vector Mammalian Two-Hybrid System used in this study provided evidence that Anxa2 directly bound to STAT3. Basing on these findings, we suppose that the direct binding of Anxa2 to STAT3 facilitates STAT3 activation in cancer cells. To confirm this hypothesis, we performed dual-luciferase reporter assay. The results demonstrated that Anxa2 enhanced the transcriptional activity of STAT3. Similarly, a previous study showed that Anxa2 binds to STAT6 and promotes its activity in prostate cancer cells [46]. These data suggest that Anxa2 not only promotes STAT3 phosphorylation but also enhances its transcriptional activity.

**Figure 8:**
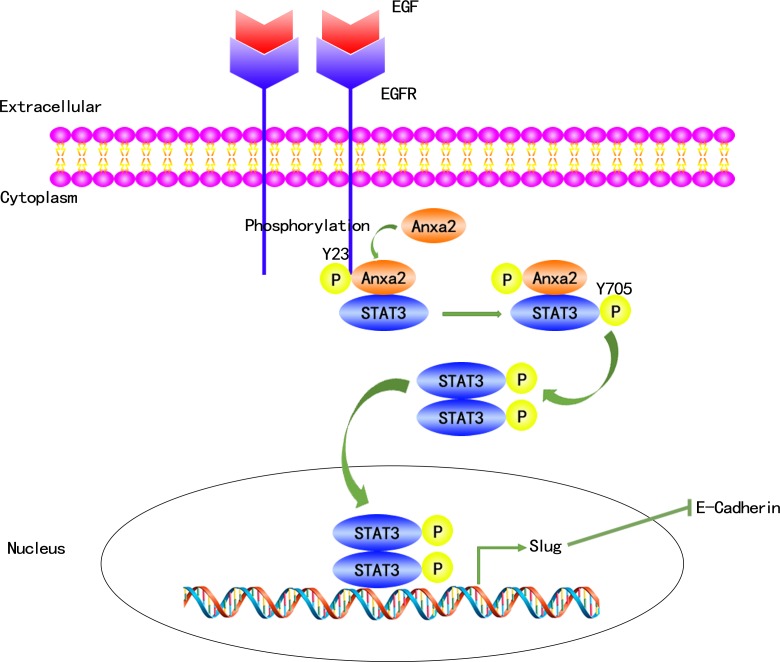
A proposed schematic model: Anxa2 mediates EGF-induced EMT in breast cancer by interacting with STAT3 The binding of EGF to EGFR leads to its autophosphorylation and activation, which, in turn, causes tyrosine phosphorylation of Anxa2, phosphorylated Anxa2 then directly interacts with STAT3 and mediates its tyrosine phosphorylation by EGFR. Phosphorylated STAT3 translocates from the cytosol to the nucleus, and upregulates the expression of EMT associated transcription factor Slug, which finally induces EMT.

In conclusion, our study demonstrates that Anxa2 expression is positively correlated with the expression of EGFR and EMT markers in breast cancer tissues and cell lines. We also showed that upregulated Anxa2 expression could be used as a predictor of poor prognosis for breast cancer patients. Moreover, we provided *in vitro* evidence that Anxa2 is required for EGF-induced EMT, as well as in the migration and invasion of breast cancer cells. Anxa2 knockdown significantly ablated pulmonary metastasis in an *in vivo* SCID mouse model of breast cancer. Anxa2 reduction also inhibited EGF-induced activation of STAT3, which is required for EGF-induced EMT. Importantly, we provide evidence that Anxa2 directly binds to STAT3 and enhances its transcriptional activity, thereby indicating that Anxa2 mediates EGF-induced EMT in a STAT3-dependent manner. These results were summarized in Figure 8. Our findings provide clinical evidence that Anxa2 is a poor prognostic factor for breast cancer and reveal a novel mechanism through which Anxa2 promotes breast cancer metastasis.

## MATERIALS AND METHODS

### Ethics statement

Investigation has been conducted in accordance with the ethical standards and according to the Declaration of Helsinki and according to national and international guidelines and has been approved by the ethics committee of Tianjin Medical University Cancer Institute and Hospital. Every patient in the study accepted the informed consent.

### Immunohistochemistry (IHC)

Paraffin-embedded invasive breast cancer specimens from 85 patients with a median follow-up time of 52 months (2-75 months) were obtained from the Tumor Bank Facility of Tianjin Medical University Cancer Institute and Hospital. Detailed procedure was reported in a previous study [47]. The antibodies of working concentration against E-cadherin and EGFR were purchased from ZSGB-BIO Company (Beijing, China). Mouse monoclonal Anxa2 and p-STAT3 (Y705) antibodies were from Santa Cruz Biotechnology. E-cadherin and EGFR staining in membrane; p-STAT3 staining in nucleus; and Anxa2 staining in membrane, cytoplasm, and nucleus were considered positive. The percentage and intensity score of stained tumor cells were determined by two independent pathologists under light microscope (Olympus Optical Co). The percentage was interpreted as follows: 0, no staining observed; 1, ≤25% of cells with positive staining; 2, 25% to 50%; 3, 50% to 75%; 4, ≥75%. In terms of the intensity score, a score of 0 referred to a negative result, 1 to a weakly positive result, 2 to a moderately positive result, and 3 to a strongly positive result. These two scores were combined and produced a final score for each slide. The scores ranged from 0 to 7, and the case was defined as high expression when the score was larger than 4.

### Cell culture and drug treatment

All the cell lines were obtained from American type culture collection and stored in our laboratory. MDA-MB-468 and MDA-MB-435 cells were cultured in DMEM/F12 medium (HyClone), and other breast cancer cell lines were cultured in RPMI-1640 (HyClone). HEK293T cells were cultured in DMEM/high glucose. All the medium contained 10% fetal bovine serum (FBS, HyClone) and 1% penicillin/streptomycin (Gibvo BRL). Cells were incubated at 37°C in a humidified atmosphere containing 5% CO_2_. The source and culture method of MCF-7/ADR cells were described in a previous study [25].

EGFR positive and epithelial phenotype breast cancer cell lines T47D and MDA-MB-468 cells were chosen to establish EGF-induced EMT models. Cancer cells were cultured routinely and serum-starved for 24 h before EGF (R&D Systems) treatment. Then the cells were cultured in 0.5 % FBS containing medium supplemented with 10 ng/mL (for T47D cells) or 100 ng/mL (for MDA-MB-468 cells) of EGF for 24h, 48h and 72h. For control group, the cells were cultured in 0.5% FBS containing medium without EGF.

### Vector construction, transfection, and stable cell lines acquirement

The Anxa2-aiming-shRNA expression vector pLKO.1 and STAT3-aiming-shRNA expression vector GV248 were obtained from Genechem Company (Shanghai, China). The targeting sequences against Anxa2 were 5′-GGTCTGAATTCAAGAGAAA-3′ (1#) and 5′-GCCAAAGAAATGAACATTC-3′ (2#). The targeting sequence against STAT3 was 5′-TGACCAACAATCCCAAGAA-3′. A sequence of similar GC content was used for negative control. Retrovirus producer cell line 293T cells were transfected with the above retroviral vectors by using Lipofectamine 2000 (Invitrogen). Virus supernatants were used to infect T47D cells. Cell lines stably expressing genes of interest were selected and maintained by 1 μg/mL of puromycin (Sigma).

Plasmids with Anxa2 mutants Anxa2Y^23A^ and Anxa2Y^23D^, which were generated by site-directed mutagenesis, were obtained in our previous study [48]. The plasmids with Anxa2 mutants were transfected to Anxa2 knockdown T47D cells, in which Anxa2 was silenced by 2# shRNA sequence targeting to noncoding region to avoid disturbance of Anxa2 mutant expression. Transfection was mediated with FuGENE HD (Promega). Subsequently, 600 μg/mL G418 (Sigma) was used to select the stable transfectants.

To study which part of Anxa2 and STAT3 interacted with each other, we established the plasmids expressing fragmental Anxa2 and fragmental STAT3. We attempted to study the two major domains of Anxa2, including N-terminal domain (1-36 amino acids) and C-terminal domain (37-339 amino acids). To conserve the two EF structures (4-90 amino acids), we divided Anxa2 into Anxa2_1-92_ and Anxa2_93-339_ fragments. For STAT3, we broke STAT3 at the linker domain and obtained the STAT3_1-475_ fragment containing N-terminal domain, coiled-coil domain, and DNA binding domain, as well as the STAT3_476-770_ fragment containing SH2 domain and transactivation domain. The total RNA of MDA-MB-231 breast cancer cells was extracted, and the synthesis of cDNA was completed by reverse transcription. The ORF of human Anxa2 and STAT3 was then amplified by regular PCR. The sequences of the primers are shown in Table S1. Plasmids expressing full-length and fragmental Anxa2 were built using PEGFP-N3 vector (Genechem Company), and the restriction enzymes BamH I and Xhol I (Thermo) were used. pCMV-C-Flag vector (Genechem company) was used to construct the plasmids expressing full-length STAT3 and two fragmental STAT3. The restriction enzymes HindIII and Sal I (Thermo) were also used. In addition, 293T cells were transfected with these plasmids in different combination using Lipofectamine 2000 (Invitrogen). After 48 h, the cells were lysed as described in co-immunoprecipitation assays below.

### Western blotting

Western blotting was performed as described previously [25, 36]. The antibodies included: mouse monoclonal antibodies against Anxa2, p-Anxa2 (Y23), Slug, and β-actin from Santa Cruz Biotechnology; rabbit monoclonal antibodies against EGFR, STAT3, p-STAT3 (Y705), Erk1/2, p-Erk, Akt, p-Akt, and Histone from Cell Signaling Technology; mouse monoclonal antibody against E-cadherin from BD Biosciences; and rabbit monoclonal antibody against Vimentin from Abcam. After incubation with the above primary antibodies overnight at 4°C, the membranes were washed with TBST three times then incubated with secondary antibodies in Odyssey blocking buffer (Gene Company) for 1 h at room temperature. The immunoreactive bands were determined by image scanning on an Image Station LI-COR Odyssey imaging system (Gene Company) and analyzed using the image software. β-actin was used as internal control.

Separation of membranous and cytoplasmic protein fractions from nuclear protein fractions

The formula of lysis buffer and method of subcellular fractionation referred to the instruction by Abcam and the method in the previous study [48]. The protein fractions were prepared for Western blot analysis. β-actin was used as internal control for membrane and cytoplasm fractions. Histone was used as control for nucleus protein fractions.

### Co-immunoprecipitation assays

The cells were seeded in 10 cm dishes and treated with 0.5% FBS-containing medium in the presence or absence of EGF for 72 h to achieve 90% confluence. Cell lysis and immonoprecipitation were performed as described in the previous study [36]. The protein concentration of supernatants of cell lysate was quantified using a BCA protein assay kit. Equal mass of protein of the EGF-treated group and the control group was immunoprecipitated with mouse monoclonal anti-Anxa2 antibody (Santa Cruz Biotechnology). The immunocomplex was captured with protein G-agarose beads (Invitrogen), and normal mouse IgG (Santa Cruz Biotechnology) was applied as negative control. The co-immunoprecipitation assay was repeated using rabbit monoclonal anti-STAT3 antibody (Cell Signaling Technology) and protein A-agarose beads (Invitrogen), and normal rabbit IgG (Santa Cruz Biotechnology) was applied as negative control. The vectors expressing full-length or fragments of Anxa2 and STAT3 were co-transfected into 293T cells in different combination by using Lipofectamine 2000 (Invitrogen) and cultured for 48 h to obtain 90% confluence in 10 cm dishes. The cell lysates were transferred to microcentrifuge tubes and centrifuged at 12,000×g for 15 min at 4°C. The supernatants were transferred to another tube and incubated with flag beads (Sigma) at 4°C overnight for further Western blot analysis.

### Immunofluorescence confocal microscopy analysis

Cells were seeded in 12-well plates containing glass coverslips, then cultured and treated with EGF as described above for 72 h. After washing with PBS three times, cells were fixed with 4% paraformaldehyde at room temperature for 10 min, permeabilized in freshly prepared 0.15% triton X-100 in PBS for 10 min, blocked in 3% BSA/PBS at room temperature for 1 h, and incubated with anti-E-cadherin (BD Biosciences), anti-Vimentin (abcam), anti-Slug (Cell Signaling Technology), anti-Anxa2 (Santa Cruz Biotechnology), anti-STAT3 (Cell Signaling Technology), and anti-p-STAT3 (Y705, Cell Signaling Technology) antibodies in a humid chamber at 4°C overnight. After repeated washing with PBS, cells were stained with anti-rabbit Alexa Fluor 488 and anti-mouse Alexa Fluor 633 conjugated secondary antibodies (Invitrogen) at room temperature for 1 h, followed by staining nucleus by DAPI. Finally, the coverslips were mounted with Mowiol-based anti-fading medium and visualized under a laser scanning confocal microscope (Leica TCS SP5).

### Wound healing assay

Cells were cultured to 100% confluence in 3.5 cm dishes, wounded using 10 μl pipette tips, washed with PBS to remove cell debris, and incubated in a medium without FBS at 37°C. The migration distances were recorded at 0, 5, 10, and 24 h. Parallel experiments with EGF treatment in the concentration of 10 ng/mL after wounding were carried out in the same way to observe the effect of EGF on the migration ability.

### *In vitro* cell invasion assay

Cell invasion assays were performed using Boyden chamber plates with 8 μm filter pores (Corning Costar Corporation) as described previously [25]. The above surface of the porous membrane of transwell inserts were coated with matrigel (BD Bioscience). Cells were trypsinized, washed with PBS, and suspended and adjusted to a concentration of 5 × 10^5^ cells/mL in FBS-free RPMI-1640 medium. Subsequently, 200 μl of cells was loaded into the upper chamber. The lower chamber was filled with FBS-free RPMI-1640 containing 10 ng/mL EGF as the chemotactic factor. After incubation at 37°C for 24 h, the invaded cells through the membrane were fixed, stained, and counted using a light microscope. All assays were performed in triplicate.

### Dual-luciferase reporter assay

CheckMate™/Flexi^®^ Vector Mammalian Two-Hybrid System and Dual-Luciferase^®^ Reporter (DLR™) Assay System were purchased from Premega. The pFN10A (ACT) vector was used to build the full-length and fragmental Anxa2 expression plasmids, and pFN11A (BIND) vector was used to build the full-length and fragmental STAT3 expression plasmids. The sequences of the primers are shown in Table S2. Different combinations of the plasmids were transfected into 293T cells by using Lipofectamine 2000 (Invitrogen), as shown in Table S3. After 48 h, the cells were lysed, and the relative value of Luc/Ren was tested by GloMax^®^20/20 (Promega).

To study the effect of Anxa2 on transcriptional activity of STAT3, 293T cells were transfected with the recombinational plasmid pSTAT3-TA-luc (D2259, Beyotime Biotechnology, Shanghai, China), which contains multiple STAT3 binding sites, pRL-TK vector, and Anxa2 fragments by using Lipofectamine 2000 (Invitrogen). The cells were cultured for 24 h and starved with FBS-free medium for another 24 h. The cells were then treated with 10 ng/mL EGF. After 24 h, the cells were lysed, and the relative value of Luc/Ren was tested by GloMax^®^20/20 (Promega) according to the manufacturer's instruction. The data were statistically analyzed.

### *In vivo* metastasis study

Five- to six-week-old female SCID mice (Vital Laboratory Animal Center, Beijing, China) were used as animal models. A total of 16 mice were divided into two groups by random sampling. One group of mice was treated with T47D/Scr cells as control, and the other group of mice was treated with Anxa2 knockdown T47D/shAnxa2 2# cells. Cells were trypsinized, washed with PBS, and suspended and adjusted to a concentration of 5 × 10^7^ cells/mL in normal saline. Approximately 200 μl of cells was injected to the tail vein of mice. Six weeks after cell injection, the mice were euthanized by intraperitoneal injection of overdose pentobarbital sodium. Lung tissues were anatomized and fixed in 4% paraformaldehyde. Tumor nodules on the surface of every lung lobe were counted, and the numbers were statistically analyzed. The lung tissues were embedded in paraffin and stained with hematoxylin-eosin (HE) for microscopy observation.

### Statistical analysis

All data were analyzed by Graphpad Prim 5.02 software. Differences in the clinicopahtological characteristics were analyzed by the chi-square test for distribution and Mann-Whitney U-test for means between groups. Cumulative incidence and survival plots were drawn using the Kaplan-Meier method. The log-rank test was used to assess the survival difference between groups. Comparison between different experimental groups was completed by using one-way ANOVA or Student's t-test, when appropriate. Quantitative data were presented as mean ± SD. *P* values less than 0.05 (two-tailed) were considered statistically significant.

## SUPPLEMENTARY MATERIAL FIGURE AND TABLES


