# Supplementary material for: Anxa2 binds to STAT3 and promotes epithelial to mesenchymal transition in breast cancer cells
Source: Oncotarget. 2015 Aug 15;6(31):30975–92. doi: 10.18632/oncotarget.5199 (PMC4741582; doi:10.18632/oncotarget.5199)
Supplement: Supplementary file 1 [file oncotarget-06-30975-s001.pdf]

Anxa2 binds to STAT3 and promotes epithelial to mesenchymal transition in breast cancer cells

Supplementary Material

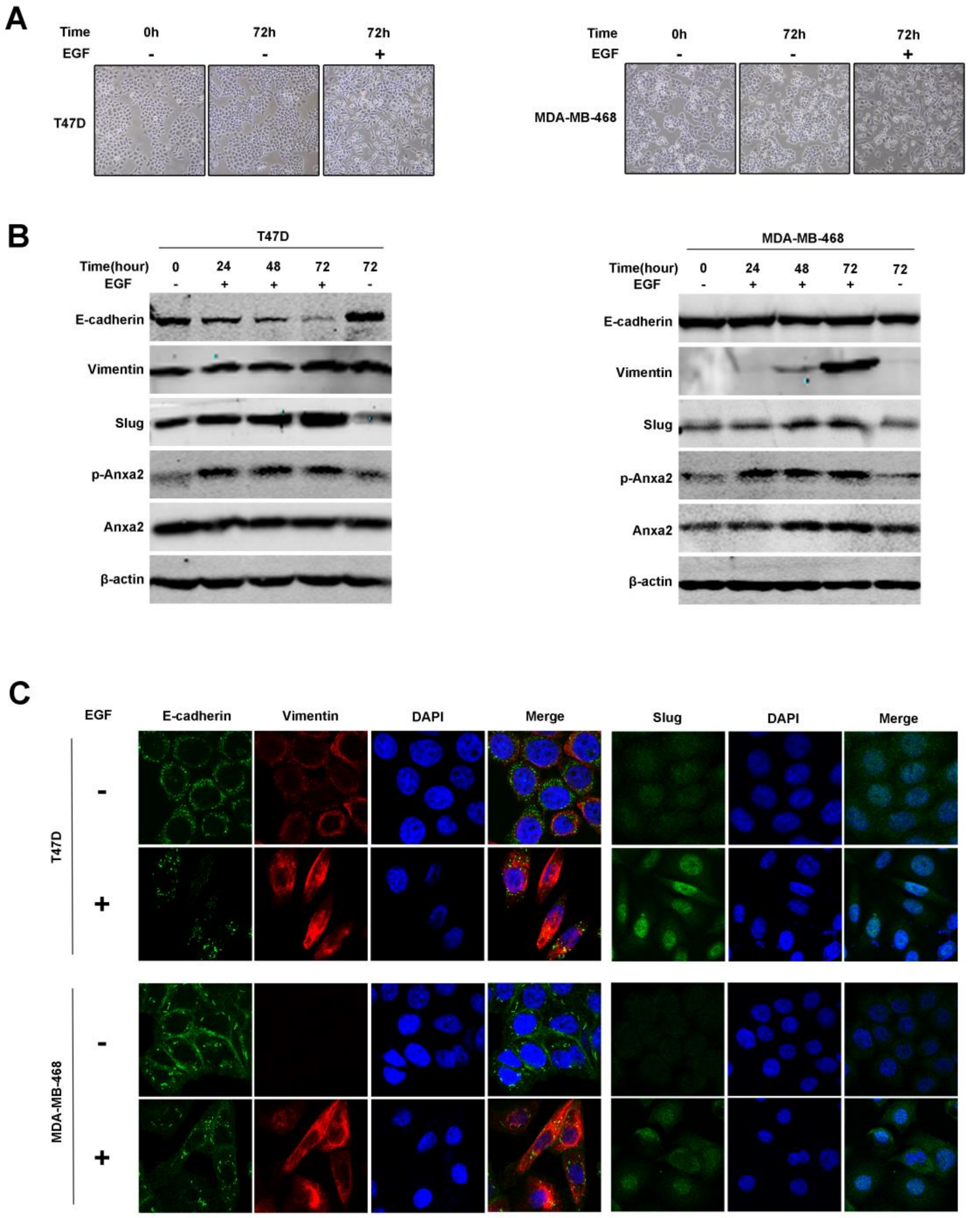

Fig. S1 Anxa2 is involved in EGF-induced EMT

A. Exposure to EGF induces morphological switch from epithelial-like to mesenchymal-like in T47D and MDA-MB-468 cells. The cells were serum-starved for 24 h, and then stimulated with or without 10 ng/mL of EGF for 72 h.

B. EGF induces EMT in T47D and MDA-MB-468 cells. Cancer cells were serum-starved for 24 h, treated with EGF for 24 h, 48 h and 72h, then the cells were harvested and analyzed by Western blotting. EGF treatment resulted in E-cadherin loss in T47D cells, Vimentin increase in MDA-MB-468 cells, and Slug upregulation in both cell lines. Anxa2 was phosphorylated at Tyr 23 during the EMT process in the two cell lines.

C. Confocal immunofluorescence microscopy analysis showed that EGF induces a significant downregulation of E-cadherin expression in the membrane and cell–cell junction, and upregulation of Vimentin, and accumulation of Slug in nucleus in T47D and MDA-MB-468 cells.

Table S1. The sequences of the primers for amplification of full-length or fragmental Anxa2 and STAT3 for co-immunoprecipitation assays

Plasmids	The sequences of primers
Anxa2FL-GFP	Forward primer:5'- CCGCTCGAGATGTCTACTGTTCACGAAAT-3'
	Reverse primer:5'- CGCGGATCCGTCATCTCCACCACACAGG-3'
Anxa2$_{1-92}$-GFP	Forward primer:5'-CCGCTCGAGATGTCTACTGTTCACGAAAT-3'
	Reverse primer:5'-CGCGGATCCAGATAAGGCTGACTTCAG-3'
Anxa2$_{93-339}$-GFP	Forward primer:5'-CCGCTCGAGATGGGCCACCTGGAGACGG-3'
	Reverse primer:5'-CGCGGATCCGTCATCTCCACCACACAGG-3'
Stat3FL-Flag	Forward primer:5'-CCAAGCTTCGATGGCCCAATGGAATCAG-3'
	Reverse primer:5'-GCAGTCGACCATGGGGGAGGTAG-3'
Stat3$_{1-475}$-Flag	Forward primer:5'-CCAAGCTTCGATGGCCCAATGGAATCAG-3'
	Reverse primer:5'-GCAGTCGACCGCCCAGGCATTTGGCAT-3'
Stat3$_{476-770}$-Flag	Forward primer:5'-CCAAGCTTCGATGTCCATCCTGTGGTAC-3'
	Reverse primer:5'-GCAGTCGACCATGGGGGAGGTAGCGC-3'

Table S2. The sequences of the primers for amplification of full-length or fragmental Anxa2 and STAT3 for dual-luciferase reporter assays

Plasmids	The sequences of primers
pFN-10A-Anxa2FL	Forward primer:5’-CTCCAGCGATCGCCATGTCTACTGTTCACG-3’
	Reverse primer:5’-AGCTTTGTTTAAACTCAGTCATCTCCACCAC-3’
pFN-10A-Anxa2$_{1-92}$	Forward primer:5’- CTCCAGCGATCGCCATGTCTACTGTTCACG-3’
	Reverse primer:5’-AGCTTTGTTTAAACTCAAGATAAGGCTGACTTC-3’
pFN-10A-Anxa2$_{93-339}$	Forward primer:5’-CTCCAGCGATCGCCATGGGCCACCTGGAGACGG-3’
	Reverse primer:5’- AGCTTTGTTTAAACTCAGTCATCTCCACCAC-3’
pFN-11A-Stat3FL	Forward primer:5’-CTCCAGCGATCGCCATGGCCCAATGGAATCAGC-3’
	Reverse primer:5’-AGCTTTGTTTAAACTCACATGGGGGAGGTAGC-3’
pFN-11A-Stat3$_{1-475}$	Forward primer:5’- CTCCAGCGATCGCCATGGCCCAATGGAATCAGC-3’
	Reverse primer:5’-AGCTTTGTTTAAACTCACGCCCAGGCATTTGGCAT-3’
pFN-11A-Stat3$_{476-770}$	Forward primer:5’-CTCCAGCGATCGCCATGTCCATCCTGTGGTAC-3’
	Reverse primer:5’- AGCTTTGTTTAAACTCACATGGGGGAGGTAGC-3’

Table S3. Different combinations of the plasmids to study the interaction between Anxa2 and STAT3 using dual-luciferase reporter assay system

Sample	ACT Vector	BIND Vector	pGL4.31(luc2P/GAL4UAS/Hygro)Vector
1	**pFN-10A-Anxa2FL**	**pFN-11A-Stat3FL**	**+**
2	**pFN-10A-Anxa2$_{1-92}$**	**pFN-11A-Stat3FL**	**+**
3	**pFN-10A-Anxa2$_{93-339}$**	**pFN-11A-Stat3FL**	**+**
4	**pFN-10A-Anxa2FL**	**pFN-11A-Stat3$_{1-475}$**	**+**
5	**pFN-10A-Anxa2$_{1-92}$**	**pFN-11A-Stat3$_{1-475}$**	**+**
6	**pFN-10A-Anxa2$_{93-339}$**	**pFN-11A-Stat3$_{1-475}$**	**+**
7	**pFN-10A-Anxa2FL**	**pFN-11A-Stat3$_{476-770}$**	**+**
8	**pFN-10A-Anxa2$_{1-92}$**	**pFN-11A-Stat3$_{476-770}$**	**+**
9	**pFN-10A-Anxa2$_{93-339}$**	**pFN-11A-Stat3$_{476-770}$**	**+**
10	**pACT Vector**	**pBIND Vector**	**+**
11	**pACT-MyoD Control**	**pBIND-Id Control**	**+**
